# Genomics for Molecular Epidemiology and Detecting Transmission of Carbapenemase-Producing *Enterobacterales* in Victoria, Australia, 2012 to 2016

**DOI:** 10.1128/JCM.00573-19

**Published:** 2019-08-26

**Authors:** Norelle L. Sherry, Courtney R. Lane, Jason C. Kwong, Mark Schultz, Michelle Sait, Kerrie Stevens, Susan Ballard, Anders Gonçalves da Silva, Torsten Seemann, Claire L. Gorrie, Timothy P. Stinear, Deborah A. Williamson, Judith Brett, Annaliese van Diemen, Marion Easton, Benjamin P. Howden

**Affiliations:** a Antimicrobial Reference and Research Unit, Microbiological Diagnostic Unit Public Health Laboratory, Department of Microbiology and Immunology, The University of Melbourne at the Peter Doherty Institute for Infection & Immunity, Melbourne, Australia; b Department of Infectious Diseases, Austin Health, Heidelberg, Australia; c Health Protection Branch, Department of Health and Human Services, Victorian State Government, Melbourne, Australia; d Doherty Applied Microbial Genomics, Department of Microbiology and Immunology, The University of Melbourne at the Peter Doherty Institute for Infection & Immunity, Melbourne, Australia; e Victorian Healthcare Associated Infection Surveillance Coordinating Centre, Melbourne, Australia; University of Iowa College of Medicine

**Keywords:** *Enterobacteriaceae*, antimicrobial resistance, carbapenemase, *Enterobacterales*, epidemiologic surveillance, molecular epidemiology, whole-genome sequencing

## Abstract

Carbapenemase-producing *Enterobacterales* (CPE) are being increasingly reported in Australia, and integrated clinical and genomic surveillance is critical to effectively manage this threat. We sought to systematically characterize CPE in Victoria, Australia, from 2012 to 2016. Suspected CPE were referred to the state public health laboratory in Victoria, Australia, from 2012 to 2016 and examined using phenotypic, multiplex PCR and whole-genome sequencing (WGS) methods and compared with epidemiological metadata. Carbapenemase genes were detected in 361 isolates from 291 patients (30.8% of suspected CPE isolates), mostly from urine (42.1%) or screening samples (34.8%). IMP-4 (28.0% of patients), KPC-2 (25.3%), NDM (24.1%), and OXA carbapenemases (22.0%) were most common. Klebsiella pneumoniae (48.8% of patients) and Escherichia coli (26.1%) were the dominant species. Carbapenemase-inactivation method (CIM) testing reliably detected carbapenemase-positive isolates (100% sensitivity, 96.9% specificity), identifying an additional five CPE among 159 PCR-negative isolates (IMI and SME carbapenemases). When epidemiologic investigations were performed, all pairs of patients designated “highly likely” or “possible” local transmission had ≤23 pairwise single-nucleotide polymorphisms (SNPs) by genomic transmission analysis; conversely, all patient pairs designated “highly unlikely” local transmission had ≥26 pairwise SNPs. Using this proposed threshold, possible local transmission was identified involving a further 16 patients for whom epidemiologic data were unavailable. Systematic application of genomics has uncovered the emergence of polyclonal CPE as a significant threat in Australia, providing important insights to inform local public health guidelines and interventions. Using our workflow, pairwise SNP distances between CPE isolates of ≤23 SNPs suggest local transmission.

## Abstract (precis)

Carbapenemase-producing *Enterobacterales* (CPE) are being increasingly reported in Australia, and integrated clinical and genomic surveillance is critical to effectively manage this threat. We sought to systematically characterize CPE in Victoria, Australia, from 2012 to 2016.

## INTRODUCTION

Carbapenemase-producing *Enterobacterales* (CPE) have emerged globally in the last two decades as a significant threat to human health, causing near-untreatable infections with high mortality rates (1–3). Comprehensive, integrated microbiological and epidemiological surveillance activities are critical to inform public health and infection control interventions but can be difficult to implement (4).

CPE have been relatively uncommon in Australia, based on data from bloodstream infection surveillance (<0.1% of Escherichia coli and 0.3% of Klebsiella pneumoniae in 2016) (5). IMP-4-producing Gram-negative bacteria (*Enterobacterales*, Pseudomonas and Acinetobacter spp.) were first reported in Australia in Melbourne, Victoria, in 2002 and subsequently also became endemic at low levels in Sydney and Brisbane, particularly affecting patients in burn wards and intensive care units (6–9). Sporadic imported cases of CPE with limited local spread emerged between 2009 and 2011 (10–12), followed by a sustained outbreak of KPC-producing *Enterobacterales* in multiple health care institutions across the state of Victoria starting in 2012 (13, 14). This KPC outbreak prompted the development of an integrated state-wide CPE surveillance system incorporating phenotypic, molecular, and genomic characterization of suspected CPE isolates coupled with detailed epidemiology, outbreak investigation, and coordinated infection control interventions and guidelines (15, 16).

In this study, we used phenotypic, molecular, and whole-genome sequencing (WGS) methods to characterize CPE collected in Victoria, Australia, from 2012 to 2016 to determine the phenotypic characteristics, antimicrobial resistance, and molecular and genomic epidemiology of local CPE, with the aim of informing future laboratory surveillance, genomic interpretations, public health interventions, and patient management.

## MATERIALS AND METHODS

### Setting.

In response to the local KPC outbreak in Victoria, Australia (population 5.92 million [17]), it became compulsory from December 2015 for all microbiology laboratories (*n* = 17) to refer suspected CPE to the state public health laboratory for molecular testing. Suspected CPE were defined as elevated meropenem MIC (≥0.5 mg/liter), reduced disc diffusion zones (meropenem, ≤24 mm [EUCAST/CLSI] [18, 19]), and/or positive phenotypic tests for carbapenemase detection (e.g., Carba NP and carbapenemase-inactivation method [CIM] testing [20, 21]), and/or carbapenemase gene detection by PCR at the local laboratory. This meropenem MIC cutoff was chosen for practical reasons, as all Victorian laboratories use Vitek 2 (AST-N246 cards; bioMérieux) for antimicrobial susceptibility testing, with the lowest reported MICs being grouped as ≤0.25 mg/liter. Statewide CPE guidelines, released in December 2015 (22), also required specific patient screening in areas of active CPE transmission and comprehensive epidemiologic data collection on all CPE cases (23) (details of CPE screening practices and microbiological methods are described in Appendix S1 in the supplemental material). Here, we describe all CPE isolates referred to the state public health laboratory from 2012 to 2016.

### Laboratory methods.

Duplicate isolates of the same species within 14 days were excluded, and nonhuman isolates were excluded. Species identification was confirmed by matrix-assisted laser desorption ionization–time of flight mass spectrometry (MALDI-TOF MS) (Vitek MS; bioMérieux). Carba NP and CIM tests were performed for isolates referred in 2016. Susceptibility testing was performed using Vitek 2 compact (AST-N246 cards; bioMérieux) and Etest (bioMérieux) for colistin, tigecycline, fosfomycin, and aztreonam. Categorical susceptibility was designated according to CLSI M-100 2016 breakpoints (19), except for colistin and tigecycline, for which EUCAST breakpoints were used (18). Carbapenemase gene detection was performed using multiplex PCR, incorporating primers for KPC, NDM, IMP, VIM, and OXA-23-, OXA-24-, OXA-48-, OXA-51-, and OXA-58-like groups, as previously described (13). KPC, NDM, IMP, and VIM alleles were further defined using Sanger sequencing. An alternative commercial PCR panel, the AusDiagnostics (Sydney, Australia) carbapenem-resistant *Enterobacteriaceae* (CRE) 16-well kit, was introduced in 2016, including additional primers for IMI and SME carbapenemases. Differences between groups were calculated using Pearson’s χ^2^ test.

### Whole-genome sequencing.

Whole-genome sequencing (WGS) was performed retrospectively on all CPE isolates from 2012 to 30 June 2015 and prospectively from 1 July 2015 to 31 December 2016. As part of a quality assurance project, WGS was also performed on all PCR-negative isolates (regardless of phenotypic results) from 1 July 2015 to 31 December 2016. Isolates were sequenced as previously described (13). Briefly, single colonies were subcultured onto horse blood agar and incubated overnight at 37°C. A single colony was placed into Gram-positive lysis buffer, followed by DNA extraction, library preparation, Illumina short-read sequencing (MiSeq or NextSeq), and assessment of sequence quality.

### Bioinformatic workflow and analysis.

To standardize and allow repeatability of the analyses of the raw sequencing data, we used the open-source software pipeline Nullarbor v2.0.20181015 (https://github.com/tseemann/nullarbor). Reads were trimmed to clip Nextera adapters and low-quality sequence using Trimmomatic v0.38 (24), *de novo* assembled using SPAdes v3.12.0 (25) via Shovill v1.0 (https://github.com/tseemann/shovill), and auto-annotated using Prokka v1.12-beta (26). The *in silico* multilocus sequence type (MLST) was determined (where a scheme exists) using the software MLST (https://github.com/tseemann/mlst). The BLAST-based Abricate search tool v0.8.10 (https://github.com/tseemann/abricate) was used to detect antimicrobial resistance (AMR) genes (minimum gene coverage, 98%; minimum gene identity, 99%) using the NCBI Bacterial Antimicrobial Resistance Reference Gene Database (27) and plasmid replicon types (minimum coverage, 98%; minimum identity, 99%) using the PlasmidFinder database (28). The pan-genome was analyzed using Roary v3.12.0 (29).

### Genomic transmission analysis.

As part of the routine workflow, new CPE isolates from each sequencing run were cross-referenced with historical isolates to determine if there were corresponding isolates of the same species, carbapenemase gene, and sequence type (ST) from other patients. When this occurred, a genomic transmission analysis was performed or updated if a previous transmission analysis had been performed. For the purposes of this study, genomic transmission analyses were performed on the 2012–2016 data set as a whole, subgrouping by species, ST, and carbapenemase gene combinations. Subgroups containing only isolates from a single patient and KPC-2 outbreak isolates were excluded, as these had been previously analyzed (13).

For the genomic transmission analysis, reads were mapped to a reference genome of the same species and ST (where possible) using Snippy v4.2 (30) to identify core single-nucleotide polymorphisms (SNPs). The closest available reference genome was determined from a preliminary Mashtree v0.50 analysis of isolates and RefSeq genomes (31), run via Pandoo (https://github.com/schultzm/pandoo) (Table S1). Phylogenetic inference was performed using IQ-TREE v1.6.5 (32) (settings: model auto-detect and ultrafast bootstrapping [1,000 replicates]). Pairwise SNP distances were compared within subgroups, and isolates were categorized by the lowest pairwise SNP distance to the nearest neighbor in the ST/subgroup. Masking of recombinant sites was performed separately (for subgroups with ≥3 isolates) using Gubbins v2.3.4 (RAxML GTRGAMMA model, 10 iterations) (33).

Genomic results were then integrated with data from concurrent epidemiologic investigations (available from late 2015). The likelihood of local CPE transmission was classified as “highly likely” (patient admissions overlapping in time and space), “possible” (admission to same hospital at same time but no direct link found), “unlikely” (admission to same hospital at different time, no direct link found), and “highly unlikely” (both patients had history of overseas travel to area of endemicity in last 4 years or were admitted to different hospitals). Pairs were designated “no data” if epidemiologic data were missing for either patient. Genomic transmission analyses were performed without prior knowledge of the results of epidemiologic investigations.

### Data availability.

Sequence data are available in the NCBI Sequence Read Archive under BioProject number PRJNA529744.

## RESULTS

A total of 1,174 suspected CPE isolates were received between 1 January 2012 and 31 December 2016 from 1,034 unique patients. Overall, 361/1,174 isolates (30.8%) were positive for known CPE genes, while 291/1,034 patients (28.1%) were positive for CPE as identified by PCR, WGS, or both methods. Fifty patients had >1 isolate collected (range, 1 to 6 isolates), including 6 patients with >1 carbapenemase gene isolated and 9 patients with the same carbapenemase gene isolated in >1 species.

### Most CPE isolates were cultured from urine or screening samples.

Of the 299 carbapenemase-positive isolates where the specimen type was documented, there were 126 (42.1%) from urine samples, 104 (34.8%) from screening rectal swabs or fecal samples, 25 (8.4%) from blood cultures, 17 (5.7%) from nonsterile sites (including wound swabs and drain fluids), 15 (5.0%) from respiratory specimens, and 12 (4.0%) from sterile sites. The median age of the patients was 68 years (interquartile range, 51 to 79 years), and 52.3% of the patients with positive CPE cultures were male.

### CIM Testing, but not meropenem MIC, reliably distinguished carbapenemase gene-positive isolates from carbapenemase gene-negative isolates.

Carba NP and CIM tests were performed in parallel on 334 isolates, including 110 carbapenemase gene-positive isolates (32.9%). CIM tests were 100% sensitive (96.9% specific), while Carba NP sensitivity was 92.7% (100% specific). All “missed” CPE isolates produced OXA carbapenemases (OXA-181 [4 isolates], OXA-48 [2], and OXA-232 and OXA-23 [1 each]). Seven isolates were CIM positive but carbapenemase gene negative by PCR, WGS, and Carba NP (six Enterobacter cloacae and one Morganella morganii).

**FIG 1:**
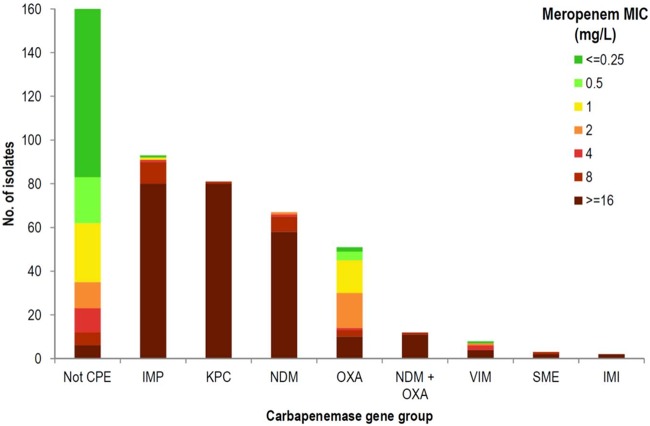
Meropenem MIC ranges of carbapenemase-positive and carbapenemase-negative isolates overlap significantly, particularly for OXA carbapenemases. The meropenem MIC is as determined by Vitek 2 automated susceptibility testing in a reference laboratory. Carbapenemase status was determined by multiplex PCR, whole-genome sequencing, or both.

Of 484 isolates, 82 (16.9%) had a meropenem MIC of ≤0.25 mg/liter when retested at the reference laboratory (below the referral criterion), and 78 (95.1%) were carbapenemase negative by PCR and/or WGS, predominantly E. cloacae complex (42.3%) (Fig. 1). Four isolates with a meropenem MIC of ≤0.25 mg/liter were carbapenemase positive by PCR (three E. coli [IMP-4, OXA-181, and OXA-23] and one K. pneumoniae [VIM-1]). All four isolates were CIM positive.

### WGS detected additional carbapenemases in PCR-negative isolates.

There were 159 suspected CPE isolates that were carbapenemase PCR negative using the initial in-house PCR assays, and these isolates underwent WGS as part of a quality control project. WGS detected additional carbapenemase genes that were not included in the in-house PCR panel in five isolates (3.2%) (two SME-2, one SME-3, one IMI-1, and one IMI-2). The presence of these carbapenemase genes was confirmed by a second PCR panel (AusDiagnostics CRE 16-well kit) with primers for these alleles. All of these isolates had high MICs to meropenem by Vitek 2 (MIC, 8 mg/liter for one SME-2 isolate; MIC, ≥16 mg/liter for other four isolates) and were positive by both Carba NP and CIM tests.

### Multiple combinations of species and carbapenemase genes are cocirculating in Victoria.

**FIG 2:**
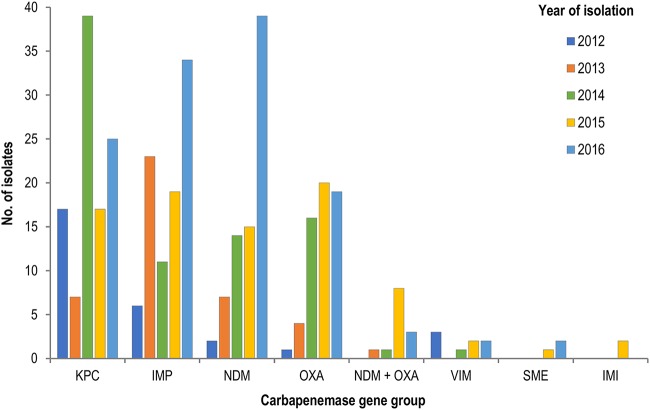
Carbapenemase gene groups isolated by year, 2012 to 2016. NDM + OXA, isolates coproducing NDM and OXA carbapenemases.

The carbapenemase alleles most commonly infecting or colonizing patients were IMP-4 (28.0% of patients), KPC-2 (25.3%), NDM-5 (11.7%), NDM-1 (8.3%), OXA-48 (8.0%), and OXA-181 (7.7%) (Fig. 2). Dual carbapenemases (in a single organism) were detected in 13 patients (4.3%), all NDM and OXA-181/OXA-232. Eight patients (2.7%) had more than one carbapenemase detected in different organisms (NDM, OXA, KPC), including one patient with four different CPE isolates.

### *Klebsiella* spp. and E. coli are the predominant CPE-containing species in Victoria.

**FIG 3:**
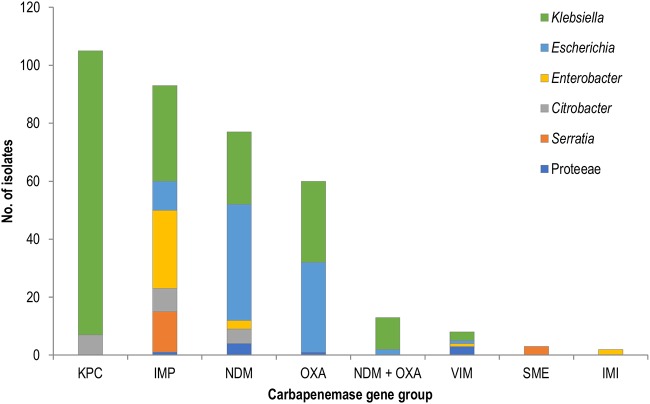
*Klebsiella* spp. and E. coli were the predominant CPE-containing species in Victoria in 2012 to 2016. *Proteeae* include *Proteus*, *Providencia*, and *Morganella* species. NDM + OXA, isolates coproducing NDM and OXA carbapenemases.

*Klebsiella* spp. accounted for over half of the CPE isolates overall (54.6%), followed by Escherichia coli (23.6%) and *Enterobacter* spp. (9.1%) (Fig. 3). NDM and OXA carbapenemases were more commonly isolated from E. coli (52.0% of NDM isolates, 36.9% of OXA isolates) and K. pneumoniae (13.7% of NDM, 14.8% of OXA), while cooccurrence of NDM and OXA enzymes occurred mainly in K. pneumoniae (11/13 NDM plus OXA isolates). KPCs were limited to K. pneumoniae except for 7 isolates (Citrobacter farmeri [6 isolates] and Citrobacter freundii complex [1 isolate]). The IMP carbapenemases (all IMP-4 except a single isolate of IMP-14) were widely spread across 8 species, including Enterobacter cloacae complex (28.0%) and K. pneumoniae (20.4%).

### Antimicrobial susceptibility testing and antimicrobial resistance genes.

**FIG 4:**
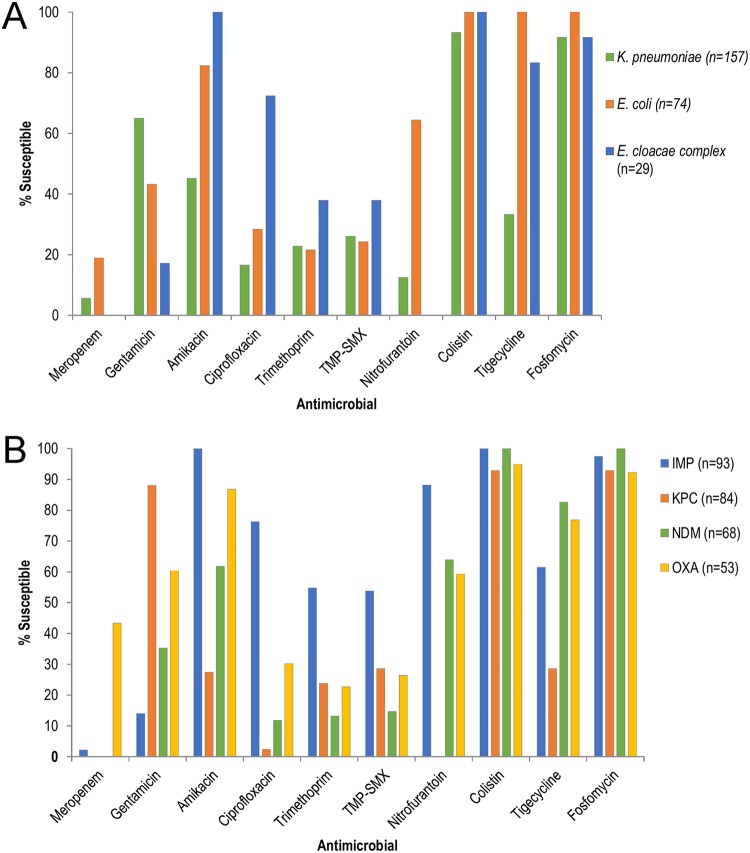
Antibiotic susceptibility of CPE isolates for the most common species and carbapenemase groups. (A) Antibiotic susceptibility by species for the three most common species (K. pneumoniae, E. coli, and E. cloacae complex). (B) Antibiotic susceptibility for the most common carbapenemase groups (IMP, KPC, NDM, and OXA). Isolates with intrinsic resistance to colistin, tigecycline, and nitrofurantoin were excluded from the analysis for these antibiotics. Susceptibility was determined by CLSI 2016 breakpoints (19), except for colistin and tigecycline (EUCAST breakpoints) (18). Nitrofurantoin results were only available for one E. cloacae complex isolate (susceptible), not shown in the figure. Refer to Table S2 for the numbers of isolates tested for each antibiotic and results for other species. TMP-SMX, trimethoprim-sulfamethoxazole.

Antimicrobial susceptibility testing (AST) results were available for 324/361 CPE isolates (Fig. 4 and Table S2); 344 had WGS available for AMR gene analysis (Table S3), and 307 had both AST and WGS. Isolates producing both NDM and OXA carbapenemases were the most resistant overall, testing nonsusceptible to most antibiotic classes except colistin and fosfomycin (5/5 isolates tested were susceptible to both).

### Testing only meropenem-resistant isolates would miss many cases of CPE.

Overall, 26/324 isolates (8.0%) were classified as susceptible to meropenem with Vitek 2 testing using CLSI clinical breakpoints (MIC, ≤1 mg/liter) and hence would not have been detected if screening was done only for carbapenem-resistant isolates (our referral criteria meropenem MIC was ≥0.5 mg/liter). OXA carbapenemase-producers had the highest meropenem susceptibility (OXA 43.4% susceptible versus others 1.1% susceptible [*P*, <0.01]; median MIC, 2 mg/liter [see Fig. 1]). No KPC, NDM, NDM plus OXA, IMI, or SME isolates tested susceptible to meropenem.

### Susceptibility was highest for colistin and fosfomycin, variable for tigecycline and aminoglycosides, and low for fluoroquinolones.

Colistin and fosfomycin were the most active antibiotics overall (125/129 [96.9%] colistin susceptible, 138/144 [95.8%] fosfomycin susceptible). *mcr* genes were not detected. Tigecycline susceptibility was low in KPC and NDM plus OXA isolates (28.6% and 20.0%, respectively) but higher in NDM, OXA, and IMP isolates (82.6%, 76.9%, and 61.5%, respectively). Amikacin susceptibility was moderate overall at 66.7% and higher in IMP- and OXA-producing isolates compared to KPC-producing K. pneumoniae (24.0% susceptible), associated with the widespread presence of *aac(6′)-Ib* genes (84.2% of KPCs). Conversely, KPC-producing isolates were more likely to be susceptible to gentamicin (88.1% susceptible). High gentamicin resistance levels in NDM-producing isolates (64.7%) were associated with ribosomal methyltransferases (*armA* or *rmt* variants, 42.5% of NDM isolates) or *aac(6′)-Ib-cr* (31.5% of NDM isolates).

Ciprofloxacin susceptibility was low (32.2% overall) except in IMP-producing isolates (76.3% susceptible). The bifunctional *aac(6′)-Ib-cr* gene encoding fluoroquinolone and amikacin resistance was found almost exclusively in NDM, OXA, and VIM isolates (including NDM plus OXA) and correlated well with ciprofloxacin resistance (43/45 [95.6%] resistant). Conversely, the widespread presence of *qnr* genes (37.8% overall) did not correlate well with quinolone resistance (*qnr* genes were present in 30.5% of ciprofloxacin resistant isolates versus 55.3% of ciprofloxacin-susceptible isolates; *P*, <0.01).

### Typing, plasmid, and genomic transmission analysis.

Of the 176 K. pneumoniae isolates, 85 (48.3%) belonged to sequence type 258 (ST258) (all KPC-2; 97.7% were linked to the local outbreak). Other common STs included ST16 (9.7%; NDM, OXA, or both) and ST15 (5.1%; mostly NDM). Overall, 26 K. pneumoniae sequence types were identified. Of the 79 CPE-producing E. coli isolates sequenced, 37 STs were detected; ST410 was the most common (17.7%). The E. cloacae isolates sequenced were polyclonal (17 STs from 32 sequenced isolates) (Table S4).

Plasmid replicon types were detected, and associations with carbapenemase genes were assessed (Table S5). Among IMP-4-producing isolates, IncA/C plasmids were common in Serratia marcescens (85.7%) and K. pneumoniae (61.1%), IncL/M plasmids were more common among E. coli and *Enterobacter* spp. (66.7% and 50.0%, respectively), and IncHI2 plasmids were most common among E. cloacae complex (61.5%). All three types have previously been described to carry the IMP-4 gene in Australia (9, 34). KPC-2-producing K. pneumoniae commonly carried IncFIB (94.6%), ColRNAI (90.2%), and IncX3 (85.7%). In contrast, NDM and OXA isolates carried a wide range of plasmid types. Plasmid replicon sequences were only rarely detected on the same contig as a carbapenemase gene. Fifteen isolates had no plasmid replicon sequences detected at these identity/coverage cutoffs, including all IMI- and SME-producing isolates (chromosomal carbapenemases).

**FIG 5:**
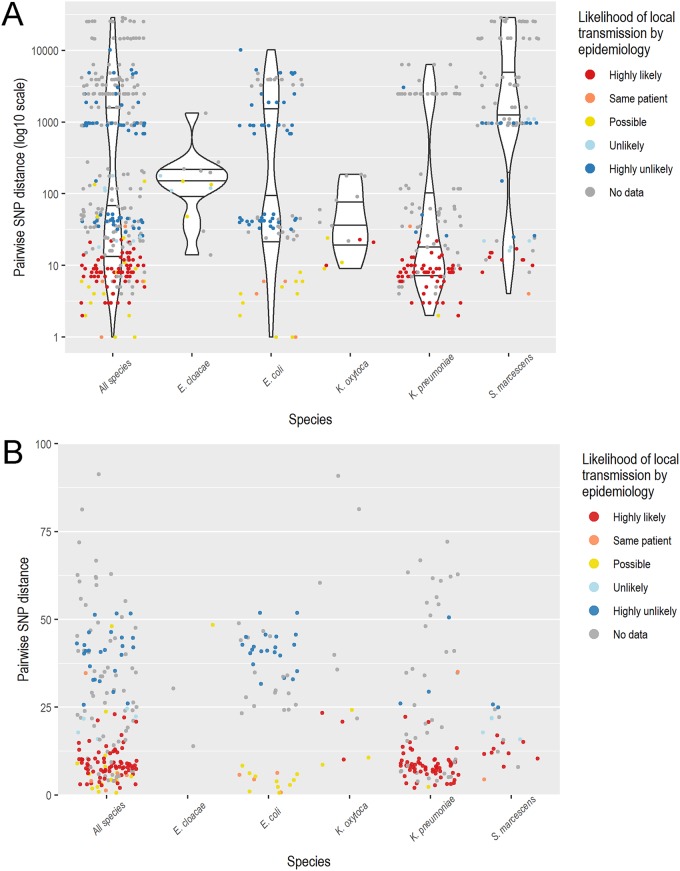
Pairwise SNP distances by species and epidemiologic data. (A) Pairwise SNP distances (log_10_ scale) plotted by species. Points are colored by the likelihood of local CPE transmission as assessed by epidemiology. SNP, single nucleotide polymorphisms (core genome). (B) Zoomed-in version of pairwise SNP distances (normal scale, maximum 100 SNPs) plotted by species. Points are colored by the likelihood of local CPE transmission as assessed by epidemiology. K. oxytoca, Klebsiella oxytoca.

Genomic transmission analyses were performed on 33 subgroups of the same species, carbapenemase gene, and ST (where available), including 131 isolates from 119 patients; subgroups included 2 to 14 isolates (median, 3 isolates) (Table S6). Pairwise SNP distances and likelihood of local transmission by epidemiology were plotted for each species (epidemiology available for 60 patients [50.4%], mostly from late 2015, when formal surveillance commenced) (Fig. 5). Pairs of isolates from the same patient (*n* = 6) had 0 to 6 pairwise SNPs, except a single patient with two ST231 OXA-232-producing K. pneumoniae isolates sampled 3 years apart (35 SNPs).

All pairs designated “highly likely” or “possible” for CPE local transmission by epidemiology had ≤23 pairwise SNPs, and 95% of these pairs had ≤20 pairwise SNPs. Conversely, all pairs designated “highly unlikely” had ≥26 pairwise SNPs, and 95% of these pairs had ≥33 pairwise SNPs. Nine patient pairs were designated “unlikely” CPE local transmission by epidemiology, including four patients from an ST114 IMP-4 E. cloacae subgroup (three of the four patients were admitted to the same hospital at similar time) and seven patients from an IMP-4 S. marcescens cluster (two patients were admitted to the same hospital as four other patients from a known transmission event but with no direct epidemiologic link; one patient was admitted to a different hospital). All subgroups (≥3 patients) with low pairwise SNPs were monophyletic with high bootstrap support (>95%), supporting the results of pairwise SNP analysis. Applying this possible SNP threshold to patients without epidemiologic data available, a further 6 subgroups (16 patients) had ≥2 nonduplicate isolates with ≤23 pairwise SNPs, suggesting possible local CPE transmission.

Masking of recombinant sites resulted in minimal changes in pairwise SNPs, except for ST478 K. pneumoniae and S. marcescens (both IMP-4). After masking recombination, two extra K. pneumoniae patients and one extra S. marcescens patient fell into the 0 to 23 SNP bracket (no epidemiologic data available for these patients). Otherwise, interpretations were unchanged (Fig. S1). For isolates classified as “highly likely” local transmission, pairwise SNP distances were generally lower for pairs of clinical isolates than for pairs of screening isolates; however, the number of clinical pairs was small (not statistically significant; see Fig. S2).

Plasmid replicon typing results were consistent with transmission analysis results in epidemiologically confirmed clusters, in that all isolates within a cluster carried the same plasmid replicon types, with the exception of the mixed NDM-5/NDM-5 plus OXA-232 cluster (K. pneumoniae ST16), where only the isolates with OXA-232 had the ColKP3-type plasmid detected, suggesting carriage of the OXA-232 gene on this plasmid.

## DISCUSSION

As in many other regions, an unexpected outbreak of a highly resistant pathogen prompted the introduction of a new public health laboratory workflow for CPE detection and characterization, incorporating genomics. Here, we investigated the molecular characteristics of CPE isolates emerging in a previously low-prevalence setting, revealing a very diverse distribution of CPE genes and host species in our state. This implies that there are multiple mechanisms for CPE introduction and transmission in our state, which are critical to understand when formulating public health intervention strategies.

This particular combination of multiple carbapenemases cooccurring in a population is unique globally, given that IMP-4 is relatively uncommon except in Australia (5, 9, 35). Previous analysis of CPE in Queensland, Australia (2009 to 2014), demonstrated the dominance of IMP-4 carbapenemases (81%), with a small number of NDM and OXA-48 group producers and no KPCs (9). Our picture of polyclonal CPE is similar to that seen in Singapore (36) and some studies from China (37) but is notably different from that seen in areas with dominant single carbapenemases, e.g., KPC in Italy (38) and the United States (39) and NDM in the United Kingdom (40) and India (41). The presence of IMP-4 across multiple species and on multiple plasmid types suggests, as others have found (9, 34, 42, 43), that the ongoing transmission of IMP-4 is predominantly due to transmission of mobile genetic elements (MGE; such as transposons and plasmids), rather than transmission of clonal bacteria of the same species. In this case, our approaches using short-read sequencing and phylogenetic analysis are limited in that they will be able to detect only a portion of IMP-4 transmission (clonal) and be unable to detect MGE transmission, either within or between patients, or from environmental organisms (excluded from this study but important for understanding transmission in many cases [7]). The future incorporation of long-read sequences promises to improve this; however, the limited accuracy of these platforms means that they are currently not suitable for transmission analysis (44).

Most patients (65.6%) had CPE identified from clinical samples alone, while 29.2% of patients had CPE identified only from screening samples. Among clinical samples, urine cultures were most common (42.1%), with only 8.4% of CPE isolates coming from blood cultures. This has implications for countries where AMR surveillance is primarily performed on blood culture isolates, such as the AGAR surveillance program (5) in Australia, and the EARS-Net surveillance program in Europe (45). Using this methodology, these programs will underestimate the overall prevalence of CPE colonization and infection in the population.

In this study, 26 CPE isolates (8%; mostly OXA-producing) had carbapenem MICs below the CLSI and EUCAST clinical breakpoints, emphasizing the importance of using lower carbapenem MICs for CPE surveillance, as recommended by EUCAST (46). In our laboratory, CIM testing performed better than Carba NP for phenotypic screening of carbapenemase producers. Parallel sequencing of carbapenemase PCR-negative isolates only detected five additional CPE isolates, all of which were CIM positive. As such, our current laboratory workflow uses CIM testing and PCR testing, with WGS for all PCR-negative CIM-positive isolates, in addition to PCR-positive isolates.

Most CPE isolates in these data set tested susceptible to colistin and fosfomycin, while susceptibilities to amikacin and tigecycline were moderate. However, the colistin and fosfomycin MICs were obtained with AST methods no longer recommended for these antibiotics and, as such, may not be accurate (47, 48). The laboratory has since moved to broth microdilution AST panels (including colistin and ceftazidime-avibactam) supplemented by agar dilution (fosfomycin) and E test (tigecycline), with susceptibility rates remaining at similar levels. In order to assist clinicians with empirical antibiotic choices, AST results have been compiled into antibiograms and made publicly available (https://biomedicalsciences.unimelb.edu.au/departments/microbiology-Immunology/research/services/microbiological-diagnostic-unit-public-health-laboratory/laboratories/epidemiology).

Analyzing genomic data to infer relationships between isolates is complex. Many variables affect the number of measured SNPs between patients, including species, sequence type, assembly quality, reference sequence selected (and relatedness to isolates being analyzed), number and diversity of isolates analyzed, time between samples, masking of recombination and/or phage elements, sequencing technology, tools employed, and SNP-calling parameters (49, 50). As such, the potential SNP “threshold” proposed by this study (pairwise SNPs below a certain number suggesting possible transmission; in this case, ≤23 SNPs) may only apply to our data set and workflows, and hence it is always important to interpret genomic data in parallel with local epidemiological data (51–53). Notably, masking recombinant sites had little effect on the pairwise SNP distances for highly related pairs by epidemiology (presumably as they had been transmitted over a short period of time), except for S. marcescens (IMP-4; no ST scheme, hence all analyzed together) and K. pneumoniae ST 478. Further work should be directed to the development of international standards for genomic transmission analysis, validation and correlation with epidemiology (e.g., standard data sets of known outbreaks and unrelated isolates), and automation of analysis to move this toward more routine incorporation into the public health laboratory workflow.

The integration of WGS into the public health laboratory for suspected CPE isolates, including rapid phenotypic testing, ≥3 WGS runs per week, semiautomation of sequence analysis, and protocolization of genomic transmission analysis (including close liaison between in-house epidemiologists and a dedicated AMR bioinformatician), has resulted in turnaround times of approximately 7 days from receipt of isolate, meaning that results can be acted upon in a clinically meaningful time frame. WGS provides a wealth of important data in addition to routine phenotypic and PCR testing, including carbapenemase allele typing (alleviating the need for a separate Sanger sequencing step), defining AMR genes (including the ability to search archived sequences for new AMR genes, e.g., *mcr*), and, perhaps most importantly for public health, transmission analysis. However, while sequencing costs are generally decreasing, significant investments in personnel (laboratory and bioinformatics), infrastructure (sequencing platforms, computing capacity, and data storage), and consumer education (clinicians and epidemiologists) are still required to implement WGS into a public health laboratory workflow (54–57).

In conclusion, we have found that a coordinated, statewide collaborative genomics and epidemiology approach to CPE has been invaluable to define the burden of CPE and direct public health and hospital infection control interventions. Further research and global cooperation are needed to optimize the application of genomics to CPE surveillance, including standardizing analyses, predicting transmission, and predicting antimicrobial resistance phenotype from genotype, in order to gain the greatest benefits from this promising technology.

## Supplementary Material

Supplemental file 1
