# Supplementary material for: Genomics for Molecular Epidemiology and Detecting Transmission of Carbapenemase-Producing Enterobacterales in Victoria, Australia, 2012 to 2016
Source: J Clin Microbiol. 2019 Aug 26;57(9):e00573-19. doi: 10.1128/JCM.00573-19 (PMC6711911; doi:10.1128/JCM.00573-19)
Supplement: Supplemental file 1 [file JCM.00573-19-s0001.pdf]

Supplementary Data: Contents

Appendix 1 – CPE screening practices in Victoria over course of study *Pages 2-3*

Table S1 – Reference genomes used for transmission analysis *Pages 4-5*

Table S2 – Antimicrobial resistance by carbapenemase gene group and species *Pages 6-7*

Table S3 – Frequency (by percentage) of selected AMR genes by carbapenemase gene group *Pages 8*

Table S4 – Multilocus sequence types (MLST) in carbapenemase-producing *K. pneumoniae, E. coli* and *E. cloacae*, 2012-2016 *Page 9*

Table S5 – Plasmid replicon types by carbapenemase gene group *Page 10*

Table S6 – Potential clusters of same species, carbapenemase and ST (where available) for genomic transmission analysis *Pages 11-13*

Figure S1 – Pairwise SNP distances by species and epidemiologic data, after masking recombinant sites *Page 14*

Figure S2 – Pairwise SNP distances by isolate type (clinical or screening) and epidemiologic data *Page 15*

References for Supplementary Data *Page 16*

Appendix 1 – CPE screening practices in Victoria over course of study

Indications for CPE screening

Prior to the release of the Victorian CPE Guidelines in December 2015, the state did not have any requirements or guidelines for CPE screening, as a low-prevalence setting; any CPE screening performed was decided on by individual healthcare networks. In general, CPE isolates were detected from clinical samples, screening of patients hospitalized overseas, or screening contacts of CPE cases (as defined by the healthcare network).

Indications for screening in the December 2015 guidelines [1] include:

- Patients directly transferred from an overseas hospital, or reporting an overnight stay in an overseas hospital or long-term residential care facility in the previous 12 months
- CPE contacts
  - Room contacts of CPE case (shared room or bathroom for ≥24 hours)
  - Ward contact of CPE case (overnight stay on ward at time where CPE transmission thought to have occurred), and
- inpatients on high-risk wards in Victorian hospitals (transplant, haematology, intensive care) during six-monthly point-prevalence survey

Completeness of CPE ascertainment

While it was not compulsory to refer isolates prior to December 2015, a retrospective review of isolates from the state of Victoria in 2015 indicated that all isolates from 2012-2015 were in fact submitted for testing at the public health laboratory.

Sample types for CPE screening

From Victorian CPE Guidelines:

In order of preference: faecal sample (preferred), rectal swab plus inguinal swab, rectal swab alone (least preferred).

Additional samples may be considered: wound swab, urine sample (if catheterized), endotracheal aspirate (if intubated), stomal specimen (if enterostomy).

Microbiological methods for CPE screening

The methods used to detect CPE from screening samples are chosen by the individual laboratories. In general, most laboratories use chromogenic agar; any colonies growing on the agar are subsequently identified (MALDI-Tof) +/- antimicrobial susceptibility testing. Since the implementation of the Victorian CPE Guidelines, any Enterobacterales growing on chromogenic media are required to be sent to the public health laboratory for further characterization. Whilst not required prior to December 2015, almost all suspected CPE isolates were sent to the public health laboratory (as confirmed by audit above).

A small number of laboratories use commercial PCR tests directly on screening samples (feces or rectal swabs); any samples with positive PCR were subsequently reflexed to culture, and forwarded to the public health laboratory for further characterization.

Table S1. Reference genomes used for genomic transmission analysis

Species	ST	Carbapenemase	Reference name/type	NCBI RefSeq accession	Reference ST	SNP distance range from isolates to reference
E. cloacae complex	24	IMP-4	*Enterobacter cloacae* strain e1087, whole genome shotgun sequence	GCF_900075335.1	738	230012-229435
	45	IMP-4	*Enterobacter cloacae* strain e840, whole genome shotgun sequence	GCF_900077875.1	45	117-127
	93	IMP-4	*Enterobacter cloacae* strain CAV1669, complete genome	GCF_001022255.1	93	1416-1418
	108	IMP-4	*Enterobacter cloacae* strain e1774, whole genome shotgun sequence	GCF_900076185.1	109	608-1300
	114	IMP-4	*Enterobacter cloacae* strain e716, whole genome shotgun sequence	GCF_900077615.1	114	229-297
	269	IMP-4	*Enterobacter cloacae* strain ND22, whole genome shotgun sequence	GCF_001662855.1	Novel ST	222649-222692
E. coli	38	OXA-48	*Escherichia coli* strain C1, complete genome	GCF_001900295.1	38	12508-18508
	167	NDM-5	*Escherichia coli* strain AR_0149, complete genome	GCF_002180055.1	167	2994-10546
	354	OXA-48	*Escherichia coli* SMS-3-5, complete genome	GCF_000019645.1	354	4736-11797
	405	NDM-5	*Escherichia coli* strain WCHEC4533, whole genome shotgun sequence	GCF_002164645.1	405	964-4958
	410	NDM-5/OXA-181	*Escherichia coli* strain Ecol_517, complete genome	GCF_002012005.1	410	1976-2998
	448	NDM-7	*Escherichia coli* strain BIDMC104, whole genome shotgun sequence	GCF_001030555.1	448	3415-4384
	648	OXA-48	*Escherichia coli* strain Ecol_881, complete genome	GCF_002012225.1	648	4213-5228
	963	OXA-23	*Escherichia coli* KTE115, whole genome shotgun sequence	GCF_000352525.1	963	7639-7647
K. oxytoca	27	IMP-4	*Klebsiella oxytoca* strain CAV1752, complete genome	GCF_001970835.1	144	29412-29446
	85	IMP-4	*Klebsiella oxytoca* strain CAV1752, complete genome	GCF_001970835.1	144	26558-26626
	novel ST	IMP-4	*Klebsiella oxytoca* 11492-1, whole genome shotgun sequence	GCF_000252915.2	36	24133-24151
K. pneumoniae	11	NDM-1	*Klebsiella pneumoniae* strain AR_0146, complete genome	GCF_002180175.1	11	90-5713
	14	NDM/OXA	*Klebsiella pneumoniae* strain KPN528 chromosome, complete genome	GCF_002156785.1	14	84-3091
	15	NDM-1	*Klebsiella pneumoniae* strain KP36 chromosome, complete genome.	GCF_001750805.1	15	2533-2588
	16	NDM-5/OXA-232	*Klebsiella pneumoniae* strain MGH165, whole genome shotgun sequence	GCF_002152595.1	16	162-2301
	16	OXA-181	*Klebsiella pneumoniae* strain MGH165, whole genome shotgun sequence	GCF_002152595.1	16	162-2301
	39	KPC-2	*Klebsiella pneumoniae* strain k1781, whole genome shotgun sequence	GCF_900086435.1	39	1557-1582
	43	OXA-181	*Klebsiella pneumoniae* strain 863_KPNE, whole genome shotgun sequence	GCF_001067575.1	43	1857-1892

Table S1 (continued). Reference genomes used for genomic transmission analysis

Species	ST	Carbapenemase	Reference name/type	RefSeq accession	Reference ST	SNP distance range from isolates to reference
K. pneumoniae continued	133	IMP-4	*Klebsiella pneumoniae* strain k722, whole genome shotgun sequence	GCF_900086095.1	133	1215-1217
	231	OXA-48	*Klebsiella pneumoniae* strain CMC_VB35168, whole genome shotgun sequence	GCF_001875525.1	231	103-3476
	231	OXA-232	*Klebsiella pneumoniae* strain CMC_VB35168, whole genome shotgun sequence	GCF_001875525.1	231	103-3476
	280	NDM-1	*Klebsiella pneumoniae* strain k1765, whole genome shotgun sequence	GCF_900086445.1	280	165-167
	359	IMP-4	*Klebsiella pneumoniae* strain FDAARGOS_89, whole genome shotgun sequence	GCF_001534855.1	359	2482-2484
	478	IMP-4	*Klebsiella pneumoniae* strain MGH134, whole genome shotgun sequence	GCF_002152435.1	478	3249-3268
P. mirabilis	NS	VIM-1	*Proteus mirabilis* strain AOUC-001, complete genome	GCF_001640985.1	NS	3206-3207
S. marcescens	NS	IMP-4	*Serratia marcescens* strain CAV1492, complete genome	GCF_001022215.1	NS	15640-30654
	NS	SME-2	*Serratia marcescens* strain UMH3 chromosome, complete genome	GCF_002220655.1	NS	34684-34689

ST, sequence type; SNP, single nucleotide polymorphisms

Table S2. Antimicrobial susceptibility by carbapenemase gene group and species

Carbapenemase gene group and species	Total no. isolates	Antibiotic, no. of susceptible isolates (%)																
		Vitek 2 testing													E-tests			
		AMC	PTZ	CRO	CAZ	FEP	MEM	GM	TB	AK	CIP	TMP	SXT	FT*	COL*	TGC*	FOS*	ATM*
**IMI**																		
Enterobacter cloacae complex	2	0	0	2 (100)	2 (100)	2 (100)	0	2 (100)	2 (100)	2 (100)	2 (100)	2 (100)	2 (100)	-	-	-	-	-
**IMP**																		
Citrobacter freundii complex	6	0	2 (33.3)	0	0	2 (33.3)	0	0	0	6 (100)	5 (83.3)	5 (83.3)	5 (83.3)	-	5/5 (100)	4/5 (80.0)	5/5 (100)	1/5 (20.0)
Klebsiella aerogenes	1	0	0	0	0	0	0	0	0	1 (100)	1 (100)	1 (100)	1 (100)	-	-	-	-	-
Enterobacter cloacae complex	24	1 (4.2)	4 (16.7)	0	0	7 (29.2)	0	1 (4.2)	0	24 (100)	18 (75.0)	8 (33.3)	8 (33.3)	1/1 (100)	10/10 (100)	8/10 (80.0)	9/10 (90.0)	5/10 (50.0)
Escherichia coli	10	1 (10.0)	5 (50.0)	1 (10.0)	1 (10.0)	6 (60.0)	1 (10.0)	2 (20.0)	1 (10.0)	10 (100)	9 (90.0)	5 (50.0)	5 (50.0)	9/10 (90.0)	4/4 (100)	4/4 (100)	4/4 (100)	3/4 (75.0)
Klebsiella oxytoca	14	0	7 (50.0)	0	0	11 (78.6)	1 (7.1)	0	0	14 (100)	14 (100)	9 (64.3)	9 (64.3)	5/5 (100)	7/7 (100)	7/7 (100)	7/7 (100)	2/7 (28.6)
Klebsiella pneumoniae	19	0	2 (10.5)	0	0	4 (21.1)	0	3 (15.8)	0	19 (100)	15 (79.0)	6 (31.6)	6 (31.6)	0/1 (0)	5/5 (100)	1/5 (20.0)	5/5 (100)	4/5 (80.0)
Proteus mirabilis	1	0	1 (100)	0	0	0	0	1 (100)	1 (100)	1 (100)	1 (100)	1 (100)	1 (100)	-	-	-	1/1 (100)	1/1 (100)
Serratia marcescens	18	0	14 (77.8)	0	0	0	0	6 (33.3)	0	18 (100)	8 (44.4)	16 (88.9)	15 (83.3)	-	-	0/8 (0)	8/8 (100)	8/8 (100)
**IMP total**	**93**	**2 (2.2)**	**35 (37.6)**	**1 (1.1)**	**1 (1.1)**	**30 (32.3)**	**2 (2.2)**	**13 (14.0)**	**2 (2.2)**	**93 (100)**	**71 (76.3)**	**51 (54.8)**	**50 (53.8)**	**15/17 (88.2)**	**31/31 (100)**	**24/39 (61.5)**	**39/40 (97.5)**	**24/40 (60.0)**
**KPC**																		
Citrobacter farmeri	5	0	0	0	0	0	0	0	0	5 (100)	0	0	0	-	3/3 (100)	2/3 (66.7)	3/3 (100)	0/1 (0)
Citrobacter freundii complex	1	0	0	0	0	0	0	0	0	0	0	0	0	-	1/1 (100)	0/1 (0)	1/1 (100)	0/0
Klebsiella pneumoniae	78	1 (1.3)	0	0	0	1 (1.3)	0	74 (94.9)	7 (9.0)	18 (23.1)	2 (2.6)	20 (25.6)	24 (30.8)	0/5 (0)	22/24 (91.7)	6/24 (25.0)	22/24 (91.7)	0/16 (0)
**KPC total**	**84**	**1 (1.2)**	**0**	**0**	**0**	**1 (1.2)**	**0**	**74 (88.1)**	**7 (8.3)**	**23 (27.4)**	**2 (2.4)**	**20 (23.8)**	**24 (28.6)**	**0/5 (0)**	**26/28 (92.9)**	**8/28 (28.6)**	**26/28 (92.9)**	**0/17 (0)**
**NDM**																		
Citrobacter amalonaticus	1	0	0	0	0	0	0	0	0	0	1 (100)	0	0	-	-	-	-	-
Citrobacter freundii complex	4	0	0	0	0	0	0	1 (25.0)	2 (50.0)	3 (75.0)	1 (25.0)	0	0	1/1 (100)	4/4 (100)	3/4 (75.0)	4/4 (100)	2/4 (50.0)
Enterobacter cloacae complex	2	0	0	0	0	0	0	1 (50.0)	1 (50.0)	2 (100)	0	1 (50.0)	1 (50.0)	-	1/1 (100)	1/1 (100)	1/1 (100)	0/1 (0)
Escherichia coli	35	0	0	0	0	0	0	12 (34.3)	9 (25.7)	23 (65.7)	4 (11.4)	3 (8.6)	4 (11.4)	21/34 (61.8)	12/12 (100)	12/12 (100)	12/12 (100)	3/12 (25.0)
Klebsiella pneumoniae	22	0	0	0	0	0	0	8 (36.4)	3 (13.6)	12 (54.6)	2 (9.1)	5 (22.7)	5 (22.7)	1/1 (100)	6/6 (100)	3/6 (50.0)	6/6 (100)	1/6 (16.7)
Morganella morganii	1	0	0	0	0	0	0	0	0	0	0	0	0	-	-	-	-	-
Proteus penneri	2	0	2 (100)	0	0	0	0	2 (100)	0	2 (100)	0	0	0	-	-	-	1/1 (100)	1/1 (100)
Providencia rettgeri	1	0	1 (100)	0	0	1 (100)	0	0	0	0	0	0	0	-	-	-	1/1 (100)	1/1 (100)
**NDM total**	**68**	**0**	**3 (4.4)**	**0**	**0**	**1 (1.5)**	**0**	**24 (35.3)**	**15 (22.1)**	**42 (61.8)**	**8 (11.8)**	**9 (13.2)**	**10 (14.7)**	**23/36 (63.9)**	**23/23 (100)**	**19/23 (82.6)**	**25/25 (100)**	**8/25 (32.0)**
**NDM + OXA**																		
Escherichia coli	1	0	0	0	0	0	0	0	0	0	0	0	0	0/1 (0)	1/1 (100)	1/1 (100)	1/1 (100)	0/1 (0)
Klebsiella pneumoniae	11	0	0	0	0	0	0	2 (18.2)	0	2 (18.2)	0	1 (9.1)	1 (9.1)	-	4/4 (100)	0/4 (0)	4/4 (100)	0/4 (0)
**NDM+OXA total**	**12**	**0**	**0**	**0**	**0**	**0**	**0**	**2 (16.7)**	**0**	**2 (16.7)**	**0**	**1 (8.3)**	**1 (8.3)**	**0/1 (0)**	**5/5 (100)**	**1/5 (20.0)**	**5/5 (100)**	**0/5 (0)**
**SME**																		
Serratia marcescens	3	0	1 (33.3)	3 (100)	3 (100)	3 (100)	0	3 (100)	3 (100)	3 (100)	3 (100)	3 (100)	3 (100)	-	-	0/1 (0)	1/1 (100)	0/1 (0)

Table S2. Antimicrobial susceptibility by carbapenemase gene group and species – Continued

Carbapenemase gene group and species	Total no. isolates	Antibiotic, no. of susceptible isolates (%)																
		Vitek 2 testing													E-tests			
		AMC	PTZ	CRO	CAZ	FEP	MEM	GM	TB	AK	CIP	TMP	SXT	FT*	COL*	TGC*	FOS*	ATM*
**OXA**																		
Escherichia coli	27	0	0	3 (11.1)	7 (29.2)	12 (44.4)	13 (48.2)	17 (63.0)	13 (48.2)	27 (100)	7 (25.9)	8 (29.6)	9 (33.3)	16/27 (59.3)	21/21 (100)	21/21 (100)	21/21 (100)	3/7 (42.9)
Klebsiella pneumoniae	24	0	0	5 (20.8)	7 (29.2)	9 (37.5)	8 (33.3)	13 (54.2)	7 (29.2)	17 (70.8)	7 (29.2)	4 (16.7)	5 (20.8)	-	16/18 (88.9)	9/18 (50.0)	15/18 (83.3)	1/3 (33.3)
Klebsiella species	1	0	0	1 (100)	1 (100)	1 (100)	1 (100)	1 (100)	1 (100)	1 (100)	1 (100)	0	0	-	-	-	-	-
Proteus mirabilis	1	0	0	1 (100)	1 (100)	1 (100)	1 (100)	1 (100)	1 (100)	1 (100)	1 (100)	0	0	-	-	-	-	-
**OXA total**	**53**	**0**	**0**	**10 (18.9)**	**16 (32.0)**	**23 (43.4)**	**21 (43.4)**	**32 (60.4)**	**22 (41.5)**	**46 (86.8)**	**16 (30.2)**	**12 (22.7)**	**14 (26.4)**	**16/27 (59.3)**	**37/39 (94.9)**	**30/39 (76.9)**	**36/39 (92.3)**	**4/10 (40.0)**
**VIM**																		
Enterobacter cloacae complex	1	0	0	0	0	0	0	1 (100)	0	1 (100)	1 (100)	0	0	-	1/1 (100)	1/1 (100)	1/1 (100)	1/1 (100)
Escherichia coli	1	0	0	0	0	0	0	1 (100)	0	1 (100)	1 (100)	0	0	1/1 (100)	-	-	-	-
Klebsiella pneumoniae	3	1 (33.3)	1 (33.3)	0	0	1 (33.3)	1 (33.3)	2 (66.7)	0	3 (100)	0	0	0	-	2/2 (100)	1/2 (50.0)	2/2 (100)	0/2 (0)
Proteus mirabilis	3	0	3 (100)	0	0	0	0	0	0	0	0	0	0	-	-	-	3/3 (100)	3/3 (100)
**VIM total**	**8**	**1 (12.5)**	**4 (50.0)**	**0**	**0**	**1 (12.5)**	**1 (12.5)**	**4 (50.0)**	**0**	**5 (62.5)**	**2 (25.0)**	**0**	**0**	**1/1 (100)**	**3/3 (100)**	**2/3 (33.3)**	**6/6 (100)**	**4/6 (66.7)**
**CARBAPENEMASE-POSITIVE TOTAL**	**324**	**3 (0.9)**	**43 (13.3)**	**16 (4.9)**	**23 (7.1)**	**61 (18.8)**	**26 (8.1)**	**154 (47.5)**	**51 (15.7)**	**216 (66.7)**	**104 (32.1)**	**98 (30.3)**	**104 (32.1)**	**55/87 (63.2)**	**125/129 (96.9)**	**84/138 (60.9)**	**138/144 (95.8)**	**40/104 (38.5)**
**Carbapenemase-negative**																		
Citrobacter freundii complex	**4**	1 (25.0)	1 (25.0)	1 (25.0)	2 (50.0)	2 (50.0)	4 (100)	4 (100)	4 (100)	4 (100)	4 (100)	4 (100)	4 (100)	1/1 (100)	-	-	-	-
Klebsiella aerogenes	**17**	1 (5.9)	2 (11.8)	0	2 (11.8)	14 (82.3)	12 (70.6)	15 (88.2)	15 (88.2)	17 (100)	16 (94.1)	14 (82.3)	15 (88.2)	0/5 (0)	-	-	-	-
Enterobacter cloacae complex	**70**	0	8 (11.4)	6 (8.6)	12 (17.1)	48 (68.6)	51 (72.9)	64 (91.4)	64 (91.4)	70 (100)	61 (87.1)	56 (80.0)	56 (80.0)	11/20 (55.0)	-	-	-	-
Escherichia coli	**22**	3 (13.6)	9 (40.9)	9 (40.9)	11 (50.0)	10 (45.5)	20 (90.9)	14 (63.6)	12 (54.6)	22 (100)	11 (50.0)	6 (27.3)	7 (31.8)	16/22 (72.7)	-	-	-	-
Hafnia alvei	**1**	0	0	0	0	0	0	1 (100)	1 (100)	1 (100)	1 (100)	1 (100)	1 (100)	1/1 (100)	-	-	-	-
Klebsiella oxytoca	**3**	2 (66.7)	2 (66.7)	2 (66.7)	3 (100)	2 (66.7)	3 (100)	3 (100)	3 (100)	3 (100)	3 (100)	3 (100)	3 (100)	2/2 (100)	-	-	-	-
Klebsiella pneumoniae	**23**	4 (17.4)	6 (26.1)	4 (17.4)	6 (26.1)	5 (21.7)	16 (69.6)	18 (78.3)	7 (30.4)	21 (91.3)	8 (34.8)	2 (8.7)	4 (17.4)	0/4 (0)	-	-	-	-
Morganella morganii	**10**	0	9 (90.0)	8 (80.0)	7 (70.0)	9 (90.0)	9 (90.0)	9 (90.0)	9 (90.0)	10 (100)	9 (90.0)	9 (90.0)	9 (90.0)	-	-	-	-	-
Proteus mirabilis	**8**	1 (12.5)	7 (87.5)	7 (87.5)	8 (100)	7 (87.5)	7 (87.5)	6 (75.0)	6 (75.0)	7 (87.5)	8 (100)	4 (50.0)	5 (62.5)	-	-	-	-	-
Serratia marcescens	**4**	0	1 (25.0)	1 (25.0)	2 (50.0)	3 (75.0)	4 (100)	4 (100)	4 (100)	4 (100)	3 (75.0)	3 (75.0)	3 (75.0)	-	-	-	-	-
**CARBAPENEMASE-NEGATIVE TOTAL**	**162**	**12 (7.4)**	**45 (27.8)**	**38 (23.5)**	**53 (32.7)**	**101 (62.3)**	**126 (77.8)**	**139 (85.2)**	**125 (77.2)**	**160 (98.8)**	**124 (76.6)**	**102 (70.0)**	**107 (66.1)**	**31 (46.3)**	**-**	**-**	**-**	**-**

Figures represent no. of susceptible isolates (%) according to CLSI M-100 2016 [2]. Isolates with intermediate susceptibility classified as non-susceptible.

Species with intrinsic resistance to the tested antibiotic (nitrofurantoin, tigecycline, colistin) were not tested and hence not included in susceptibility totals

*Nitrofurantoin, colistin, tigecycline, fosfomycin and aztreonam results not available on all isolates, figures represent no. of susceptible isolates out of no. tested (%). “-”, not tested.

Table S3. Frequency (by percentage) of selected AMR genes by carbapenemase gene group

AMR genes detected by WGS (No. of isolates)	Frequency (%) of AMR genes detected by carbapenemase gene group							
	IMP (89)	KPC (101)	NDM (73)	NDM + OXA (13)	OXA (55)	VIM (8)	All CPE (344)	CP neg (155)
**Extended-spectrum beta-lactamases (ESBLs)**								
bla_{CTX-M}	19.1	2.0	54.8	100	67.3	50.0	**32.8**	**19.4**
ES-bla_{SHV}	21.3	9.9	5.5	0	9.1	0	**11.0**	**5.2**
bla_{CMY}	5.6	1.0	26.0	15.4	32.7	12.5	**13.4**	**3.2**
**Aminoglycoside resistance genes**								
rmt/armA	0.0	1.0	42.5	100	9.1	37.5	**15.4**	**0.6**
aac(3)-IId	79.8	6.9	19.2	23.1	21.8	37.5	**32.0**	**0**
aac(6')-Ib	0.0	84.2	5.5	0	3.6	0	**26.5**	**0.6**
aac(6')-Ib-cr	1.1	0.0	31.5	31	36.4	25	**14.5**	**15.5**
**Other resistance genes**								
dfr	39.3	76.2	83.6	92	72.7	100.0	**67.7**	**31.0**
qnr	65.2	6.9	41.1	46	43.6	62.5	**37.8**	**16.8**
sul	100.0	77.2	86.3	92	76.4	100	**84.9**	**30.3**
tet	37.1	7.9	41.1	77	45.5	50.0	**32.0**	**12.3**

ES-bla_{SHV}, extended-spectrum (ESBL) bla_{SHV} genes; bla_{CMY}, AmpC-type ESBL gene; *rmt* and *arm,* ribosomal methyltransferases; *aac*, aminoglycoside acetyltransferase; *dfr,* dihydrofolate reductase (trimethoprim resistance); *qnr,* quinolone resistance (plasmid-mediated); *sul,* sulfonamide resistance; *tet,* tetracycline resistance. *aac(3)-IId* encodes gentamicin resistance; *aac(6')-Ib* encodes amikacin resistance; *aac(6')-Ib-cr* encodes both amikacin and fluoroquinolone resistance.

Isolates with ≥1 copy or variant of the same gene class were only counted once. 'All CPE' column includes other carbapenemase groups not listed here (IMI, SME). 'CP neg', carbapenemase-negative isolates (by PCR, WGS or both).

Table S4. Multilocus sequence types (MLST) in carbapenemase-producing *K. pneumoniae, E. coli* and *E. cloacae*, 2012-2016

K. pneumoniae (*n*=176 isolates)		*E. coli* (*n*=79 isolates)		*E. cloacae* (n=30 isolates)	
ST	No. of isolates	ST	No. of isolates	ST	No. of isolates
**258**	85	410	14	93	4
**16**	17	405	7	114	4
**15**	9	648	6	269	3
**231**	7	354	5	24	2
**11**	5	10	3	45	2
**147**	5	38	3	108	2
**478**	5	156	3	90	1
**530**	4	448	3	94	1
**14**	3	69	2	113	1
**39**	3	167	2	116	1
**359**	3	224	2	152	1
**13**	2	963	2	175	1
**43**	2	58	1	252	1
**48**	2	73	1	418	1
**133**	2	95	1	477	1
**280**	2	127	1	780	1
**307**	2	131	1	-	3
**340**	2	154	1		
**17**	1	155	1		
**27**	1	189	1		
**101**	1	205	1		
**323**	1	227	1		
**383**	1	258	1		
**395**	1	349	1		
**1122**	1	359	1		
**1583**	1	361	1		
**-**	8	394	1		
		401	1		
		457	1		
		617	1		
		940	1		
		1434	1		
		1702	1		
		4108	1		
		4450	1		
		5135	1		
		-	3		

ST, sequence type; ‘-‘, no matching ST available

Table S5. Plasmid replicon types by carbapenemase gene group

Plasmid replicon types	Frequency (%) of plasmid replicon types detected by carbapenemase gene group						
	IMP (89)	KPC (101)	NDM (73)	NDM + OXA (13)	OXA (55)	VIM (8)	All CPE (344)
**IncA/C**	43.3	2.0	6.8	0	3.6	50.0	15.1
**IncFIA**	1.1	0	31.5	69.2	43.6	0	16.5
**IncFIB**	17.8	91.1	34.2	76.9	25.5	37.5	46.4
**IncFII**	10.0	7.9	17.8	53.8	9.1	0	12.2
**IncHI1**	0	0	11.0	23.1	5.5	12.5	4.3
**IncHI2**	27.8	5.9	4.1	0	0	25.0	10.4
**IncI1**	1.1	0	8.2	0	5.5	0	2.9
**IncL/M**	41.1	0	0	0	12.7	0	12.8
**IncN**	10.0	0	1.4	0	0	25.0	3.5
**IncQ**	0	0	11.0	15.4	23.6	37.5	7.5
**IncR**	11.1	7.9	20.5	61.5	7.3	25.0	13.6
**IncX**	0	78.2	32.9	76.9	30.9	0	37.7
**Col**	2.2	82.2	23.3	84.6	41.8	0	39.4

Plasmid replicon types detected by BLAST search tool (abricate) using PlasmidFinder database (minimum gene coverage 98%, minimum gene identity 99%).

Bracketed numbers under carbapenemase genes denote total number of sequenced isolates for each group.

Table S6. Potential clusters of same species, carbapenemase and ST (where available) for genomic transmission analysis

Species	ST	Carbapen-emase	No. isolates	No. patients	Pairwise SNP group (no. patients)	Changes masking recomb'n†	Likelihood of local transmission by epidemiology	Agreement between genomics and epidemiology?
***E. cloacae* complex**	24	IMP-4	2	2	>100 SNPs	NP	NA	-
	45	IMP-4	2	2	24-50 SNPs	NP	NA	-
	93	IMP-4	2	2	0-23 SNPs	NP	NA	-
	108	IMP-4	2	2	>100 SNPs	NP	NA	-
	114	IMP-4	4	4	24-50 SNPs (2)<br>>100 SNPs (2)	-	Unlikely (no direct patient links, but both admitted to same hospital in past at different times)	Insufficient data
	269	IMP-4	3	3	>100 SNPs	-	NA	-
E. coli	38	OXA-48	2	2	>100 SNPs	NP	NA	-
	167	NDM-5	2	2	>100 SNPs	NP	Highly unlikely (overseas acquisition)	Yes
	354	OXA-48	5	5	24-50 SNPs (3)<br>>100 SNPs (2)	-	Local transmission highly unlikely for one patient pair (46 SNPs), both thought to be acquired overseas. Others NA	No
	405	NDM-5	6	4	>100 SNPs	-	Highly unlikely (overseas acquisition)	Yes
	410	NDM-5/ OXA-181	12	11	0-23 SNPs (7)<br>24-50 SNPs (2)<br>>100 SNPs (2)	-	Five patients with low pairwise SNPs (1-8 SNPs) currently under investigation (isolated from same hospital, although history of overseas travel). Other patient pairs thought unrelated (overseas acquisition)	Partial
	448	NDM-7	2	2	>100 SNPs	NP	NA	-
	648	OXA-48	3	3	>100 SNPs	-	Highly unlikely (overseas acquisition)	Yes
	963	OXA-23	2	2	24-50 SNPs	NP	NA	-
K. oxytoca	27	IMP-4	3	3	0-23 SNPs (2)<br>24-50 SNPs (1)	-	NA	-
	85	IMP-4	4	4	51-100 SNPs (3)<br>>100 SNPs (1)	-	NA	-
	novel ST††	IMP-4	4	4	0-23 SNPs (4)	-	Highly likely - local transmission event declared for 3 most closely-related isolates (9-21 SNPs). Other patient possible (11-24 SNPs)- admitted to same facility, but no direct contact	Yes

Table S6, continued. Potential clusters of same species, carbapenemase and ST (where available) for genomic transmission analysis

Species	ST	Carbapen-emase	No. isolates	No. patients	Pairwise SNP group (no. patients)	Changes masking recombt'n†	Likelihood of local transmission by epidemiology	Agreement between genomics and epidemiology?
K. pneumoniae	11	NDM-1	5	4	>100 SNPs	-	NA	-
	14	NDM-1/ OXA-232	3	3	51-100 SNPs (2)<br>>100 SNPs (1)	-	Highly unlikely (overseas acquisition)	Yes
	15	NDM-1	7	5	0-23 SNPs (5)<br>>100 SNPs (1)	-	NA	-
	16	NDM-5/ OXA-232	13	13	0-23 SNPs (13)	-	Highly likely – local transmission event declared for 11/13 of highly-related isolates; other pair (distant to first group) NA	Yes
	16	OXA-181	2	2	>100 SNPs	NP	Highly unlikely (overseas acquisition)	Yes
	39	KPC-2	3	3	24-50 SNPs	-	Highly unlikely (overseas acquisition)	No
	43	OXA-181	2	2	51-100 SNPs	NP	NA	-
	133	IMP-4	2	2	0-23 SNPs	NP	Highly likely – local transmission event declared	Yes
	231	OXA-48	2	2	0-23 SNPs	NP	NA	-
	231	OXA-232	4	3	0-23 SNPs (2)<br>24-50 SNPs (1)	-	NA	-
	280	NDM-1	2	2	0-23 SNPs	NP	Unlikely related by epidemiology (patients in different hospitals from same network within 1 week)	No<br>Possible laboratory error?**
	359	IMP-4	3	2	24-50 SNPs	-	NA	-
	478	IMP-4	6	6	0-23 SNPs (3)<br>24-50 SNPs (3)	0-23 SNPs (5)<br>24-50 SNPs (1)	Highly likely (3 most closely-related isolates, 5-22 SNPs), local transmission event declared. Other isolates NA	Yes
P. mirabilis	NS	VIM-1	3	2	24-50 SNPs	-	NA	-

Table S6, continued. Potential clusters of same species, carbapenemase and ST (where available) for genomic transmission analysis

Species	ST	Carbapen-emase	No. isolates	No. patients	Pairwise SNP group (no. patients)	Changes masking recombt'n†	Likelihood of local transmission by epidemiology	Agreement between genomics and epidemiology?
S. marcescens	NS	IMP-4	14	13	0-23 SNPs (9)<br>>100 SNPs (4)	0-23 SNPs (10)<br>51-100 SNPs (1)<br>>100 SNPs (2)	4 patients highly likely; local transmission event declared.<br>3 patients unlikely (no direct epi link found to cluster); one patient 17-26 SNPs to cluster, other 2 patients >100 SNPs to cluster. After masking recombination, all 3 patients 4-6 SNPs to cluster.<br>Other patients NA	Partial
	NS	SME-2	2	2	>100 SNPs	NP	NA	-

ST, sequence type; NS, no MLST scheme available; NA, no epidemiologic data available, '-' no comparison as no epidemiologic data available.

†Changes in allocation of patients to pairwise SNP groups after masking of recombinant sites. '-' no change, 'NP' not performed (unable to mask recombination with Gubbins if <3 isolates).

††Note: novel ST, identical at all 7 alleles

*Note: three patients had isolates in two different clusters (patient 1, *E. coli* ST 410 (NDM-5, OXA-181) and *K. pneumoniae* ST 14 (NDM-1, OXA-232); patient 2, *E. coli* ST 354 (OXA-181) and ST 648 (OXA-4); patient 3, *K. oxytoca* ST 27 and *E. cloacae* ST 108, both IMP-4)

** Patients were hospitalised at different hospital sites (served by same hospital laboratory) at the same time, with no previous admissions, and no epidemiologic evidence of overlap. Isolates were referred to reference laboratory in same batch from same hospital laboratory; same results were obtained on repeat testing from isolates stored at hospital laboratory. Epidemiologic investigations concluded that hospital laboratory error was most likely.

Figure S1. Pairwise SNP distances by species and epidemiologic data, after masking recombinant sites

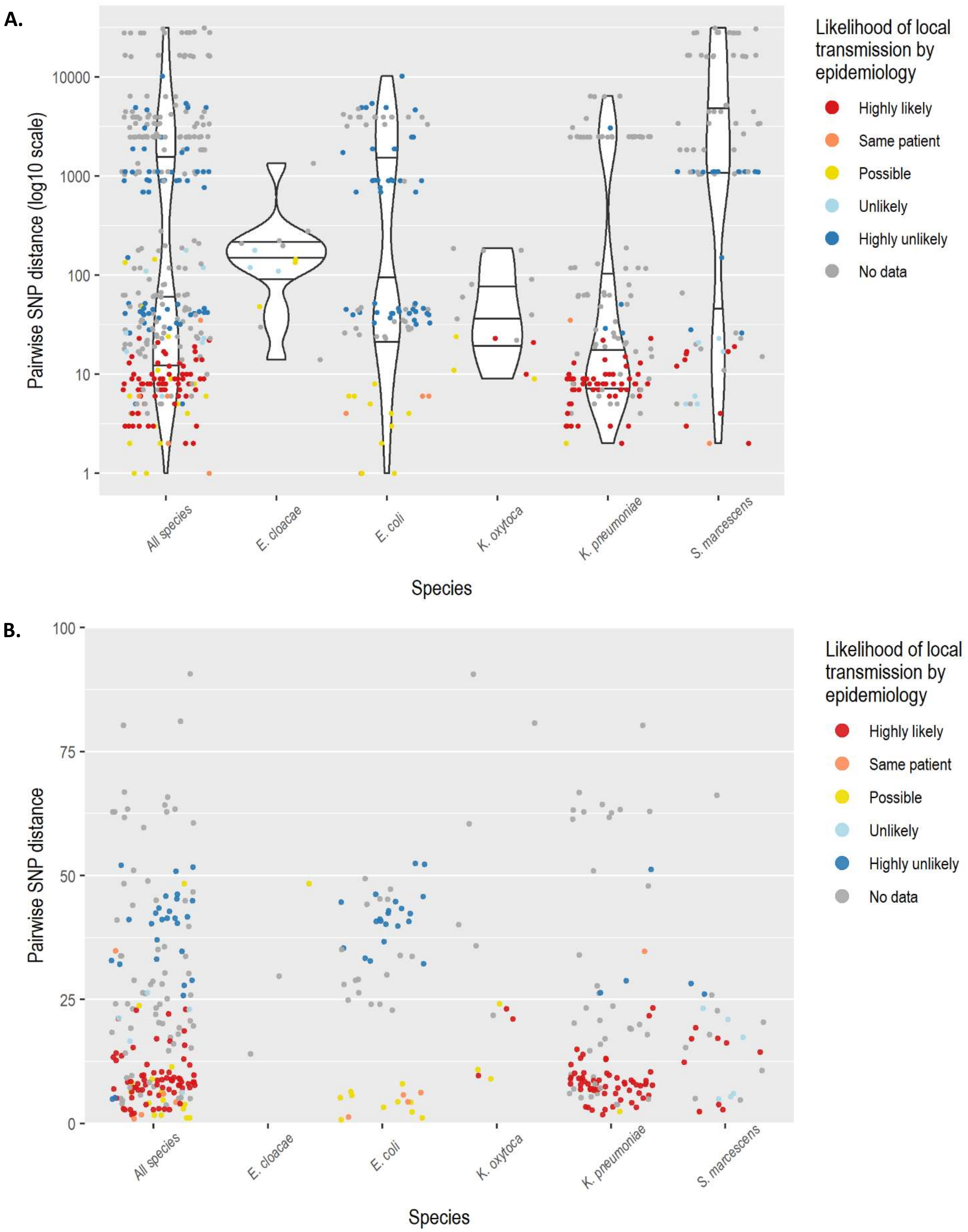

Panel A. Pairwise SNP distances (log_{10} scale) plotted by species, after masking of recombinant sites (Gubbins). Points colored by likelihood of local CPE transmission, as assessed by epidemiology.

Panel B. Zoomed-in version of pairwise SNP distances (normal scale, maximum 100 SNPs) plotted by species, point colored by likelihood of local CPE transmission.

Images prepared using same code as Figure 5 in manuscript (before masking recombination).

SNP, single nucleotide polymorphisms (core genome).

Figure S2. Pairwise SNP distances by isolate type (clinical or screening) and epidemiologic data

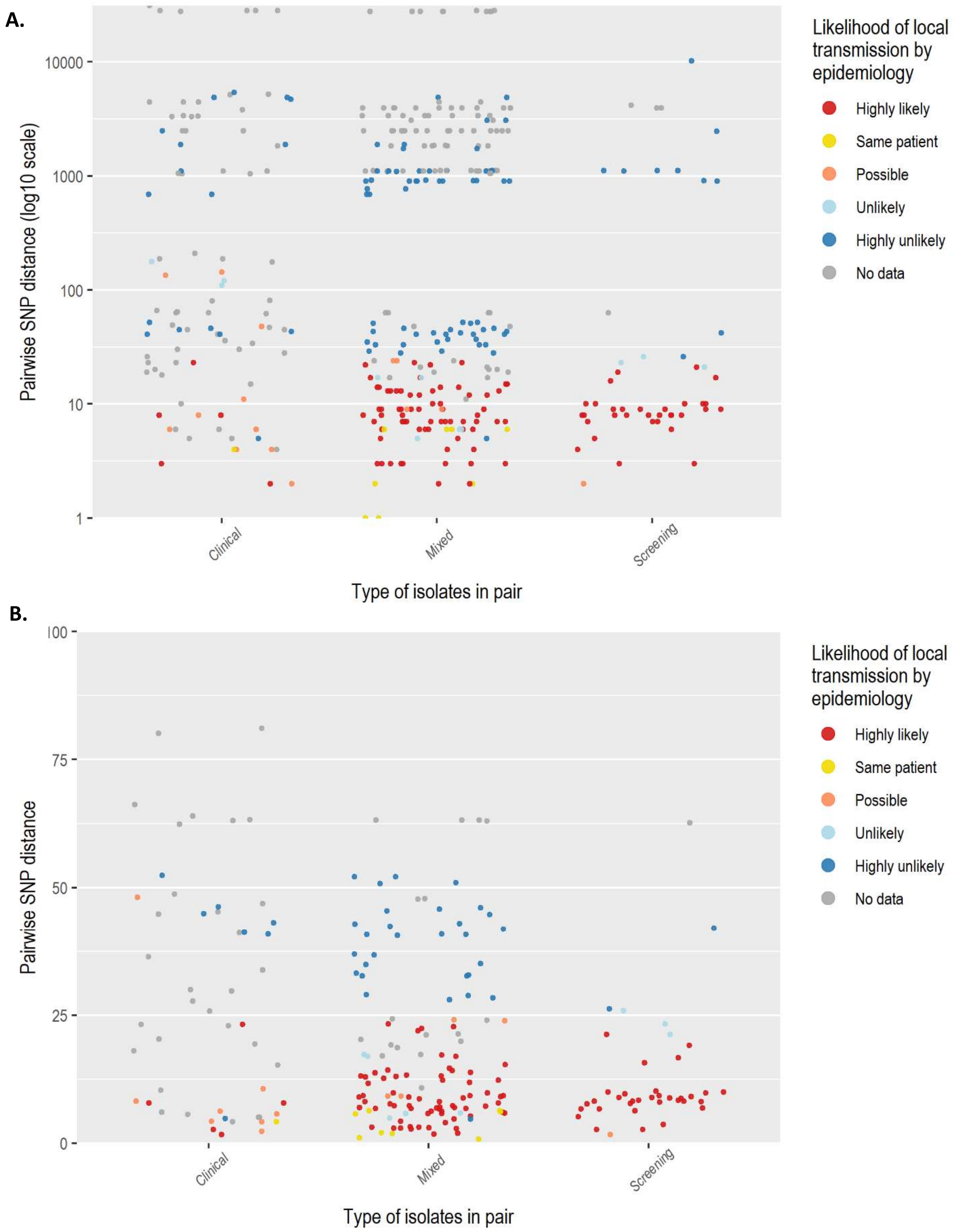

Panel A. Pairwise SNP distances (log_{10} scale) plotted by isolate pair type (both clinical, both screening, or mixed; excludes unknown species types), after masking of recombinant sites (Gubbins). Points colored by likelihood of local CPE transmission, as assessed by epidemiology.
Panel B. Zoomed-in version of pairwise SNP distances (normal scale, maximum 100 SNPs) plotted by isolate pair type, point colored by likelihood of local CPE transmission.
SNP, single nucleotide polymorphisms (core genome).

References

1. Department of Health and Human Services Victoria, *Victorian guideline on carbapenemase-producing Enterobacteriaceae (for health services)*, Department of Communicable Disease Prevention and Control, Editor. 2015, Victorian Government: Melbourne.
2. Clinical & Laboratory Standards Institute, *Performance standards for antimicrobial susceptibility testing (26th ed.) CLSI supplement M100*. 2016.
